# Risk-Adjusted Mortality: Problems and Possibilities

**DOI:** 10.1155/2012/829465

**Published:** 2012-03-15

**Authors:** Daniel Shine

**Affiliations:** Department of Medicine, NYU Langone Medical Center, 550 First Avenue, New York, NY 10016, USA

## Abstract

The ratio of observed-to-expected deaths is considered a measure of hospital quality and for this reason will soon become a basis for payment. However, there are drivers of that metric more potent than quality: most important are medical documentation and patient acuity. If hositals underdocument and therefore do not capture the full “expected mortality” they may be tempted to lower their observed/expected ratio by reducing “observed mortality” through limiting access to the very ill. Underdocumentation occurs because hospitals do not recognize, and therefore cannot seek to confirm, specific comorbidities conferring high mortality risk. To help hospitals identify these comorbidities, this paper describes an easily implemented spread-sheet for evaluating comorbid conditions associated, in any particular hospital, with each discharge. This method identifies comorbidities that increase in frequency as mortality risk increases within each diagnostic grouping. The method is inductive and therefore independent of any particular risk-adjustment technique.

## 1. Introduction

Risk of death in a hospitalized patient—and therefore the number of deaths expected in a hospital—is usually calculated using demographic and coded diagnostic and procedural information. A variety of private companies, trade organizations, and government agencies have developed mathematical models for calculating expected mortality; most of them employ roughly the same data set and use similar techniques of logistic regression [1–6]. The ratio between actual and expected deaths is widely considered to reflect quality of care, and as hospital performance data circulate ever more widely on the internet, this ratio has become a metric of increasing prominence [7, 8].

Soon risk-adjusted mortality will be of importance also to hospital revenue. Last year, the Center for Medicare and Medicaid Services (CMS) announced plans to withhold from each hospital a percentage of payments derived from diagnosis-related groupings (DRGs). These funds will be kept in a national pool and redistributed using indicators of quality and patient satisfaction chosen by CMS. A hospital may lose all of its withheld payments or receive well over twice the amount withheld. Observed-to-expected mortality in three DRGs is scheduled in 2014 to become one of the factors determining the percentage returned [9].

As hospitals attempt to maximize payment, they will take straightforward steps to increase compliance with those processes identified by CMS as quality related. They will also create initiatives to improve patient satisfaction. However, it is more difficult to conceive what specific measures a hospital might employ to decrease observed-to-expected mortality overall. With an important financial stake but no available strategy to protect that stake, hospitals will approach observed-to-expected mortality with concern.

To patients as well as insurance companies, there is undeniable appeal in a metric that purports to compare life-saving prowess between hospitals. Many will want to choose a hospital based perhaps largely on such a metric. It is therefore important both to make certain that the metric is fairly applied and to understand the limits on our ability to identify institutions that prevent death.

### 1.1. Observed Deaths

Observed deaths, the numerator, are usually an unavoidable result of end-stage or sudden and overwhelming illness. These deaths, and not the relatively few cases for which quality issues are determinative, mainly populate the numerator of observed/expected mortality. Because marginally preventable deaths are relatively uncommon, numerator variations tend to be more responsive to the acuity of a hospital's patients than to the quality of its care. Every patient who dies increases numerator more than denominator because expectation of death is never 100%. The inevitable effect of increasing acuity is therefore an unwanted increase in the ratio. On the other hand, surviving low-acuity patients always increase denominator more than numerator because expectation of death is never 0%. The result is a desirable decrease in the ratio.

Enhancing quality of care is of course the intended strategy—improving observed/expected mortality by decreasing the “observed” numerator selectively among those patients whose survival is problematic but within reach. These are a small minority of numerator cases, however, while lowering institutional acuity broadly improves both numerator and denominator. Lower acuity also decreases the number of extremely long-stay patients, perhaps the most important driver of another closely watched hospital parameter, average length of stay.

Avoiding very ill patients or transferring them before they die may be mathematically a more effective way to decrease mortality ratio and practically easier to implement than a quality improvement effort with no very clear focus. The use of observed/expected mortality as a quality marker and a reimbursement multiplier may, therefore, threaten to limit hospital access for the very ill.

### 1.2. Expected Deaths

Not only institutional acuity but also choice of risk adjustment paradigm influences the denominator (“expected deaths”). Current methods of risk adjustment have been shown to predict death with variable accuracy and to disagree with each other substantially and often [10–12].

Variability in these calculations of mortality risk arises from the intrinsic difficulty in assessing severity of the principal diagnosis itself, particularly using administrative data that lack clinical detail. ICD-9 disease categories underlying all risk adjustment capture subtle differences in etiology but characterize severity less well, mainly by appending comorbidities. Yet severity of the principal diagnosis is of the first importance in predicting death, and mortality is also influenced by demographic and psychosocial factors, such as access to care and treatment setting [13, 14]. Like disease severity, the effect of these factors is not well captured by ICD-9.

Describing interactions among the intrinsic severity of a principal diagnosis, psychosocial factors, and the large number of possible comorbid conditions is the major mathematical problem of risk adjustment. Opportunities are many for excluding important variables and associations, for under- and overfitting, and for model instability due to variable colinearity. Inclusion of late-occurring, virtually death-defining diagnoses (asystole or cardiac arrest, e.g.,) as predictors of death can artificially enhance the apparent predictive power of risk adjustment models.

An important additional reason for poor model precision is that documentation practices vary among hospitals. Those with “sicker” patients have been shown to be often overcoded [15]. Equally problematic is the observation that patients who die tend as a group to be undercoded [16]. Whether extracted during record review or sent to databases automatically after coding, comorbid diagnoses are derived from documentation in the medical record; it is their assigned weights that largely determine expected risk of death in most adjustment methodologies. Both over-and undercoding can lead, therefore, to inaccurate risk assessment. Perhaps the most important kind of overcoding is failure to distinguish between complications and comorbidities [17]. CMS now requires hospitals to designate comorbidities that were present on admission (POA); however, compliance with this requirement is not complete even when a POA determination is easily made. The result can be risk adjustment that “charges” to the patient medical conditions actually caused by the hospital, resulting in overestimation of expected mortality [18, 19].

Undercoding arises from a failure to document or code those few comorbidities within each diagnostic grouping that specify a substantially increased risk of death. Within any risk-adjustment model, each comorbidity has a coefficient relating that comorbidity to the likelihood of death only in a particular DRG (or other grouping) and often only in the company of other particular comorbidities, but that coefficient is rarely large. Clinicians and coders cannot easily pick out these large-coefficient comorbidities because there are too many combinations of risk level, grouping, and comorbidity to keep track of. In addition, the majority of institutions purchase risk adjustment services that are proprietary; the general logic of their method is available, but specific determinants of risk are usually not.

A hospital, therefore, may not easily identify characteristic comorbidities that contribute heavily to risk in that particular hospital's common clinical groupings. It is desirable to find the comorbid conditions most relevant to risk of death for any particular illness and using any risk-adjustment method. Also useful to individual hospitals would be a more general list of those conditions that often contribute to local mortality risk in a range of locally common illnesses.

## 2. Methods

### 2.1. Data Sources

We approached this problem using a simple spreadsheet applied to our risk-adjusted data in two different risk adjustment methodologies, 3M (St. Paul, MN) and University Healthcare Consortium (UHC, Chicago IL).

Downloads came from our decision support system (McKesson HBOC, San Francisco, CA) for the 3M method and from UHC. The Institutional Review Board approved this approach and waived the requirement for individual patient consent in analyzing this deidentified patient data.

### 2.2. Data Manipulations

Risk adjustment data from these sources were downloaded onto separate Excel spreadsheets (Microsoft, Redmond, WA). Spreadsheet manipulation was divided into two phases: first, counting the number of instances of each comorbidity in each clinical grouping (APRDRG for 3M or base MSDRG for UHC) and second, identifying in each clinical grouping those comorbidities whose prevalence increased markedly between contiguous (and across all four) risk categories. These changes in prevalence were measured as slopes (Figure 2).

Discharge level data was downloaded onto the left hand side of eight spreadsheets, one for each of the four risk adjustment levels in each risk adjustment methodology. Separate columns contained for each discharge the groupings APRDRG, MSDRG, and base MSDRG (UHC), and up to 50 coded comorbid conditions. Each sheet was sorted by the grouping used in that risk adjustment methodology so that discharges in the same grouping were in contiguous rows.

**Figure 1:**
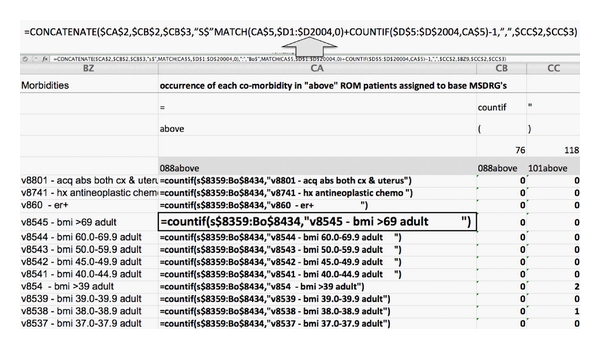
Organization of the right side of the UHC risk level “above” spreadsheet to count occurrences of comorbidities in each of the 100 commonest base MSDRGs.

Counting comorbid conditions was achieved by creating a grid on the right of each spreadsheet defined by the hundred commonest groupings (displayed along a row as column headings) and (to the left of these displayed in a column as row headings) all the comorbidities that occurred once or more often. Cells at the junction of a particular comorbidity row and a particular grouping column were programmed to calculate the number of times this comorbidity occurred in discharges belonging to that grouping (within the level of risk to which the current spreadsheet was assigned). This is shown in Figure 1.

In order to count comorbidities in these cells, a text statement was developed for each cell that would, later, be converted to a calculating formula. The text statement is shown in Figure 1 (above the arrow) as it was written in the formula bar for the highlighted cell. This statement was assembled from concatenated fragments such as “=” and “)” (also shown in Figure 1 below the formula bar), whose spreadsheet location was specified in the formula bar text. As the text statement was entered into each cell, adjusted to reflect the particular row and column of that cell, the cell processed “concatenate” commands and cell references in the text, displaying the simpler statement shown in the highlighted cell in Figure 1. This simplified statement, copied and “pasted as value,” was next converted to a formula in all grouping columns by using the Excel command “replace” to change “=” to “=”. This apparently purposeless maneuver actually forces each cell to reexamine the text statement and then treat it as a formula.

With the text statement changed to a formula, each cell now directs that occurrences of the comorbidity named in the current row be counted among the spreadsheet's 50 columns devoted to comorbidities. However, this comorbidity count is to be limited to the range of rows containing patients in the grouping named by the current column. For example, suppose in Figure 1 that the current cell in the “Above” risk level spreadsheet is the one highlighted at the junction of MSDRG 88 (the grouping naming the current column) and “V 8545 BMI > 69 adult” (the comorbidity naming the current row). The highlighted cell is instructing that all instances of “v8545” be counted in the 50 comorbidity columns (“s” through “bo”). However, counting must occur only in the row range that contains discharges in the “Above” risk level of MSDRG 88 (rows 8434 through 8545).

The first of these rows was found in this clinical grouping column (MSDRG in Figure 1) using Excel's “match” function with the grouping name and risk level. Finding the last row was achieved by adding to the “match” result the number of rows containing that name (using the “countif” function).

In this manner, the number of occurrences of each comorbidity among patients belonging to each grouping was calculated in a matrix format. Occurrences in individual groupings or across any number of groupings could then readily be summed at each level of risk. Prevalence was reported as the number of comorbidity occurrences divided by the number of discharges, either in a single grouping or across a range of groupings.

**Figure 2:**
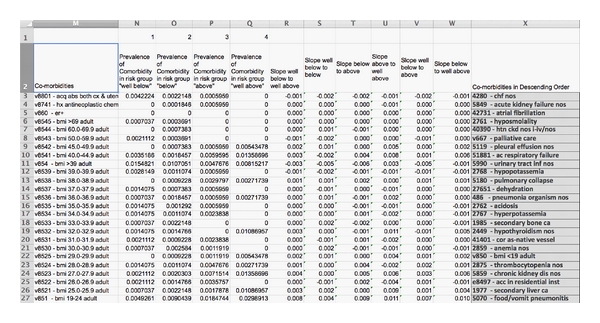
Slope of comorbidity prevalence among the commonest 100 base MSDRGs in all four levels of risk in the UHC methodology. Comorbidities are shown in descending order of slope magnitude.

The second step in spreadsheet manipulation was to evaluate changes in the prevalence of each comorbidity, either within a particular grouping or across the commonest groupings, as risk levels increase. This was achieved by transferring the list of calculated comorbidity prevalences for each risk level into a new spreadsheet and determining rate of change in these values (slope) for each comorbid condition between risk levels from minor to extreme (APRDRG) or well below to well above (UHC). Those morbidities with the largest slopes were then identified by sorting (Figure 2).

## 3. Results

The 100 commonest groupings (accounting for about 90% of discharges) were examined all together in both risk adjustment methods, and the ten commonest (about 30%) were examined from the perspective of individual grouping.

Shown in Table 1 are comorbidities with the largest 25 slopes across all risk levels for the 100 commonest groupings in the two risk adjustment systems. The two methods shared 18 of the 25.

## 4. Discussion

Using this inductive method, we identified comorbid conditions that were associated with increases in morbidity risk category at a particular institution. It appears that the method, applied here to two, could be used for many risk- adjustment paradigms. Hospitals now focus on educating doctors about the importance of documenting comorbid conditions that increase the complexity and reimbursement of common DRGs. Perhaps equally important in maximizing revenue and hospital reputation will be educating doctors to identify and document those conditions that increase risk of death. Risk may be measured by different insurers using different methods, making an inductive approach to assessing the results of any method easier for a hospital than trying to duplicate each method.

An important limitation of this approach is that it cannot distinguish diagnoses *defining* increased risk from those *associated *with increased risk. The first is clearly a subgroup of the second, raising questions not about sensitivity but the specificity of this method. An important topic for future study is whether the likelihood increases with its slope that a particular comorbidity *places* rather than *accompanies* patients into a higher risk category.

Knowledge of comorbidities associated with risk has, of course, the potential both to improve and to undermine precision in risk adjustment. Just as it is likely for a hospital to undercode comorbid conditions that it does not know to be important, so the possibility of overcoding arises when a hospital knows or suspects which diagnoses will increase reported risk of death (and therefore improve its observed/expected mortality). On the other hand, it can be argued that hospitals, which already suffer regulatory and financial consequences when they are found to overcode, should not also, through ignorance of the basis for risk, be systemically encouraged to underestimate their expected mortality [20, 21]. In assessing the effect of this or other methods that may be developed to deconstruct risk adjustment paradigms, it is important to measure the effect both on recognition and documentation of conditions that would otherwise be missed and on reporting of conditions that are not in fact present. As risk-adjusted mortality grows to be more important a measure, these studies will hopefully be performed.

Finally, it should be noted that widespread use of any technique that accurately increases documentation and coding as a specific and rapid response to the results of risk adjustment may in turn affect the process of risk adjustment itself. For example, a comorbidity more widely reported because it is identified as enhancing risk will enhance risk less. Shared knowledge of risks between adjusters and clinicians may well create a dynamic relationship with unknown effects.

## Figures and Tables

**Table 1:** Comorbidities with the greatest slopes across all four risk levels in the methods used by UHC and 3M.

Comorbidity	3M	UHC
584.9: acute renal failure NOS	×	×
428.: CHF unspecified	×	×
518.81: acute respiratory failure	×	×
599.: urin tract INFEC/bacteriuria	×	×
V66.7: encounter for palliative care	×	×
486: pneumonia, organism NOS	×	×
57.: food/vomit pneumonitis	×	×
427.31: atrial fibrillation	×	×
414.1: cornry atheroscelersis native	×	×
511.9: pleural effusion NOS	×	×
276.1: Hyposmolality	×	×
518.: pulmonary collapse	×	×
276.2: acidosis	×	×
E849.7: ACCID in resident INSTIT	×	×
43.9: HY KID NOS W CR KID I-IV	×	×
198.5: secondary malig neo bone	×	×
285.9: anemia NOS	×	×
995.92: severe sepsis	×	×
25.: DMII WO COMP NT ST UNCNTR	×	
785.52: septic shock	×	
272.4: hyperlipidemia NEC/NOS	×	
V15.82: history of tobacco use	×	
77.3: decubitus ulcer, low back	×	
41.9: hypertension	×	
38.9: septicemia NOS	×	
276.8: hypopotassemia		×
276.51: dehydration		×
244.9: hypothyroidism NOS		×
276.7: hyperpotassemia		×
585.9: chronic kidney dis NOS		×
287.5: thrombocytopenia NOS		×
197.7: secondary liver Ca		×
